# An unusual presentation of intraocular tuberculosis in a monocular patient: clinicopathological correlation

**DOI:** 10.1186/s12348-016-0118-8

**Published:** 2016-11-25

**Authors:** Kanika Aggarwal, Aniruddha Agarwal, Shobha Sehgal, Suryaprakash Sharma, Nirbhai Singh, Kusum Sharma, Ramanuj Samanta, Alessandro Invernizzi, Aman Sharma, Vishali Gupta

**Affiliations:** 1 Advanced Eye Center, Department of Ophthalmology, Post Graduate Institute of Medical Education and Research (PGIMER), Sector 12, Chandigarh, 160012 India; 2 Department of Histopathology, Post Graduate Institute of Medical Education and Research (PGIMER), Chandigarh, India; 3 Department of Medical Microbiology, Division of Mycobacteriology, Post Graduate Institute of Medical Education and Research (PGIMER), Chandigarh, India; 4 Eye Clinic, Department of Biomedical and Clinical Science “Luigi Sacco”, Luigi Sacco Hospital, University of Milan, Milan, Italy; 5 Department of Internal Medicine, Division of Rheumatology, Post Graduate Institute of Medical Education and Research (PGIMER), Chandigarh, India

**Keywords:** Intraocular tuberculosis, Enhanced depth imaging optical coherence tomography, Mycobacteria, Histopathology, Dalen Fuchs spots, Autofluorescence, Enucleation

## Abstract

**Background:**

Lack of uniform diagnostic criteria often poses a challenge in the diagnosis and management of tubercular uveitis. The index case describes an unusual presentation of tubercular panuveitis initially misdiagnosed as sympathetic ophthalmia, where the appropriate diagnosis was made using various imaging and laboratory investigations.

**Results:**

A 52-year-old Indian woman underwent multimodal imaging, extensive clinical and laboratory work-up, and analysis of microbiological and histopathological specimens. At presentation, her best-corrected visual acuity (BCVA) was 20/30 in OD and no perception of light in OS. Ocular examination revealed multiple grayish-yellow choroiditis lesions resembling Dalen-Fuch’s nodules, vitritis, and disc edema. Diagnosis of sympathetic ophthalmia was made and patient treated with intravenous and oral corticosteroids and immunosuppressive therapy. After an initial favorable response, the lesions progressively increased with worsening of vitritis. Due to worsening of chorioretinal lesions which were atypical for sympathetic ophthalmia, further investigations were performed that revealed positive tuberculin skin test and contrast-enhanced computerized tomography chest showed calcified mediastinal lymph nodes. Enucleation of OS confirmed acid-fast bacilli on Ziehl-Neelsen staining, tubercular granulomas on histopathology, and positive polymerase chain reaction. Anti-tubercular therapy and oral steroids were started with good healing response.

**Conclusions:**

Tubercular uveitis may have protean clinical manifestations. Thorough clinical evaluation and molecular/histopathological evaluation helps in establishing the diagnosis and the institution of appropriate therapy.

## Findings

### Introduction

Intraocular tuberculosis (IOTB) is a form of extrapulmonary TB and has been reported to be the most common etiology of infectious uveitis in TB-endemic regions [1–3]. It can present with protean clinical manifestations with varied anatomic, morphological, and imaging characteristics. However, the paucibacillary nature of disease in the eye makes the confirmation of diagnosis by standard microbiological methods such as demonstration of *Mycobacterium tuberculosis* organisms on smear microscopy or culture from ocular samples difficult. Nucleic acid amplification techniques such as multi-targeted polymerase chain reaction (PCR) have greatly enhanced the sensitivity and specificity of detection from ocular tissues [4–6].

Accurate diagnosis of IOTB often needs a multimodal approach with a combination of a comprehensive clinical evaluation and targeted laboratory investigations. The index study describes an unusual case where the diagnosis of IOTB was confirmed with the help of microbiological and histopathological evaluation of the enucleated specimen from the fellow non-seeing eye.

### Case report

**Fig. 1:**
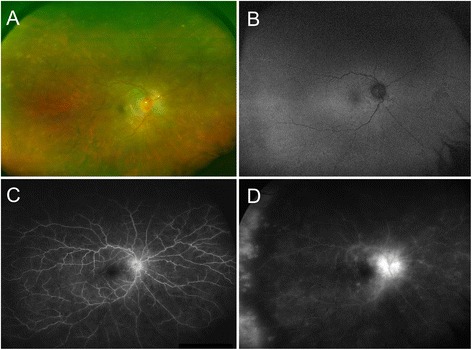
**a** Fundus photograph of the right eye at the time of initial presentation shows presence of vitritis, optic disc edema, and multiple faint grayish-yellow subretinal lesions. **b** Fundus autofluorescence imaging shows the presence of subtle alterations in the autofluorescene in the areas corresponding to the grayish-yellow choroidal lesions. Fluorescein angiography image of the right eye shows disc hyperfluorescence and multiple hypofluorescent lesions scattered in all quadrants in the early frames (**c**) which became iso/hyperfluorescent in the late frames (**d**)

**Fig. 2:**
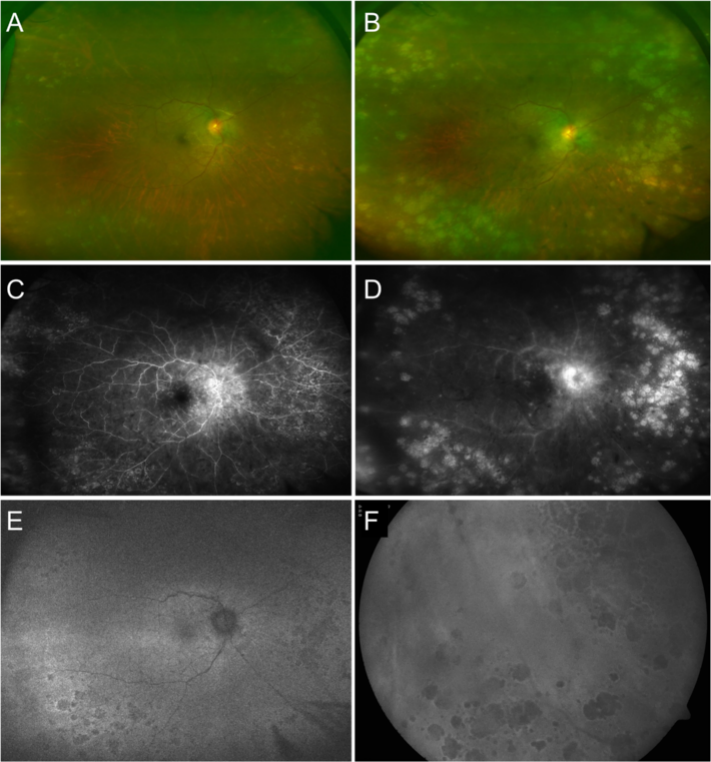
Fundus photograph of the right eye after the initial healing response with interval improvement in the vitritis and disc edema. Multiple grayish lesions can be seen in the retinal periphery which appear healed (**a**). There was subsequent increase in number and size of these grayish-yellow choroidal lesions along with worsening of vitritis and recurrence of disc edema after 8 weeks of immunosupression (**b**). Fluorescein angiogram (FA) (in the early frame) showed optic disc hyperfluorescence and multiple areas of transmission hyperfluorescence scattered throughout the retinal periphery which were suggestive of healed lesions along with multiple hypofluorescent lesions suggestive of active choroidal lesions (**c**). Late frames of FA revealed disc hyperfluorescence along with hyperfluorescent lesions suggestive of active disease along with vessel wall hyperfluorescence suggestive of active vasculitis (**d**). Fundus autofluorescence image (with the ultra-wide field fundus camera) shows the presence of hypo-autofluorescent lesions with hyper-autofluorescent borders in the retinal periphery (**e**) as well as in the peripapillary region. These hypo-autofluorescent lesions with hyper-autofluorescent borders are also appreciated on conventional color fundus camera (**f**). The presence of such hypo-autofluorescent lesions did not favor the diagnosis of sympathetic ophthalmia raising the clinical suspicion of an alternate diagnosis

A 52-year-old woman of Indian origin presented in November 2015 with complaints of decreased vision in right eye (OD) for the past 1 month. She had a history of trauma to the left eye (OS) with a stone in 2013. At the time of presentation, the best-corrected visual acuity (BCVA) was 20/20 in OD and no perception of light in OS. On ocular examination, anterior segment examination of OD revealed 1+ cells and 1+ flare, and granulomatous keratic precipitates (KPs) in the anterior chamber. Posterior segment examination showed presence of round-to-oval outer retinal/choroidal grayish-yellow lesions in all the quadrants with presence of peripapillary fluid (Fig. 1). The OS was phthisical and the details of the anterior segment could not be appreciated. Fluorescein angiography (FA) (Fig. 1c, d) showed the presence of multiple hypofluorescent lesions in the early frames which progressively became iso- to hyperfluorescent with disc hyperfluorescence in the late frames. Indocyanine green angiography (ICGA) could not be performed since the patient declined. Enhanced-depth imaging optical coherence tomography (EDI-OCT) scan passing through the macula showed normal foveal contour with peripapillary retinal thickening. The subfoveal choroidal thickness was increased (326 μm). Given the monocular status and past history of trauma to the fellow eye, a clinical working diagnosis of sympathetic ophthalmia was made. The patient was initiated on therapy with intravenous methylprednisolone (1 gm/day for 3 days) followed by oral corticosteroids (1 mg/kg prednisone) along with oral azathioprine (2 mg/kg). One week after initiation of therapy, there was a resolution of the peripapillary fluid with interval improvement in the fundus lesions (Fig. 2a).

**Fig. 3:**
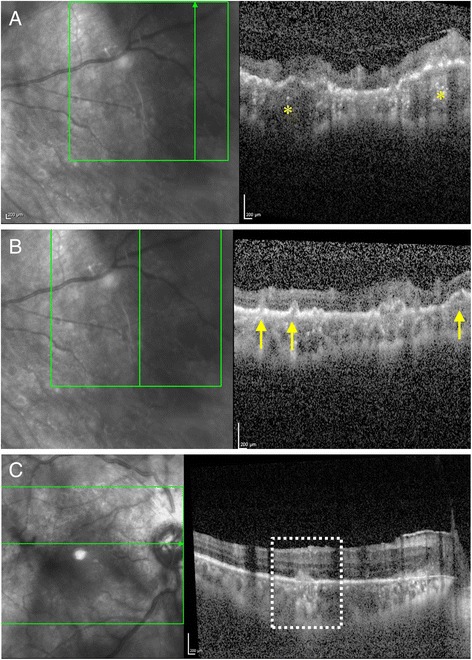
Enhanced-depth imaging optical coherence tomography (EDI-OCT) vertical line scan passing through the choroidal lesions shows retinal pigment epithelial (RPE) elevations with homogenous hyporeflectivity in the underlying choroid suggestive of choroidal granulomas (*yellow asterisks*) (**a**). Such choroidal granulomas were multiple and present throughout the retinal midperiphery and periphery. EDI-OCT vertical line scans passing through another region of active choroiditis lesions in the periphery shows multiple RPE bumps and elevations (*yellow arrows*) (**b**) and outer retinal hyperreflective deposits seen in the macular region (*dotted square*) (**c**)

However, on her follow-up visit at 3 months, the fundus lesions had increased in number and size with recurrence of vitritis (Fig. 2b). Her BCVA deteriorated to 20/80 with recurrence of vitritis. At this stage, FA showed early hyperfluorescence corresponding to the peripheral retinochoroidal lesions with progressive hyperfluorescence in the late frames (Fig. 2c, d). In addition, there were multiple small hypofluorescent lesions which were more posterior in location that became iso- to hyperfluorescent in the late frames. On fundus autofluorescence (FAF) imaging, there was presence of hypo-autofluorescence in the areas of chorioretinal lesions (Fig. 2e, f). EDI-OCT showed characteristic outer retinal and RPE lesions which resembled Dalen-Fuch’s nodules. However, there were deeper choroidal lesions that appeared atypical for sympathetic ophthalmia (Fig. 3). Since presence of chorioretinal lesions on EDI-OCT and FAF alterations were unusual clinical manifestations for sympathetic ophthalmia, additional investigations were performed to rule out the possibility of infectious etiologies and masquerades in this patient.

Laboratory tests revealed a positive tuberculin skin test (18 × 18 mm) and QuantiFERON TB Gold® test. Contrast-enhanced computerized tomography of the chest revealed multiple calcified lesions in both the upper lobes of the lung with small mediastinal lymph nodes (not amenable to endobronchial ultrasound transbronchial needle aspiration (EBUS-TBNA). Whole body positron emission tomography (PET) scan revealed fluorodeoxyglucose (FDG) avid subcentimetric mediastinal lymph nodes and FDG avid patchy consolidatory changes in both lung upper lobes likely to be post-granulomatous changes. An FDG avid lesion was present in the left orbit.

**Fig. 4:**
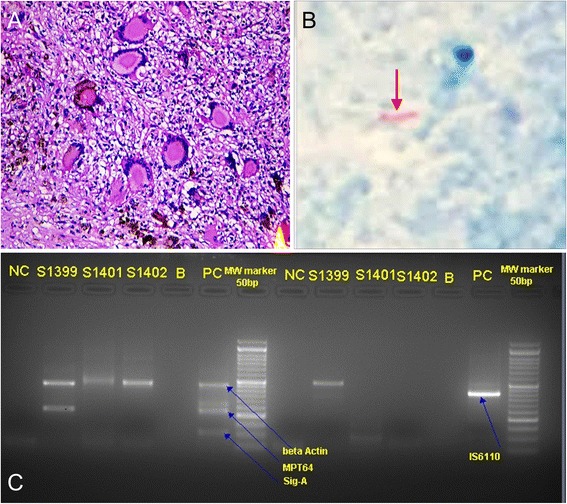
Hematoxylin and Eosin staining of the histopathological section revealed presence of multiple epitheloid cell granulomas surrounded by multinucleated giant cells with central caseous necrosis suggestive of tubercular (TB) etiology (**a**). Ziehl-Neelsen staining of the histopathological section shows the presence of acid-fast bacilli (*red arrow*) (**b**). Polymerase chain reaction conducted on the fluid aspirate from the enucleated eyeball was positive for two TB specific primers (IS6110 and MPT64) (**c**)

**Fig. 5:**
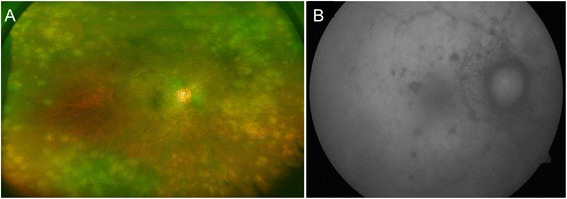
Fundus photograph of the right eye at the final follow-up visit showed resolution of optic disc edema and decrease in vitritis with multiple grayish lesions in the periphery in various stages of healing (**a**). Fundus autofluorescence image shows the presence of peripapillary and macular hypofluorescent lesions suggestive of healing tubercular choroiditis (**b**)

In order to rule out the presence of lymphoma and to determine if the etiology of the fundus lesions was related to TB, the patient was advised enucleation of the non-seeing eye (OS). The patient agreed for the procedure and enucleation of OS was performed. Histopathological examination revealed presence of multiple choroidal granulomas with caseous necrosis (Fig. 4a, b). TB PCR was positive from the vitreous fluid sample of the enucleated globe (Fig. 4c). Ziehl-Neelsen staining performed on the histopathology specimen showed presence of numerous acid-fast bacilli (Fig. 4b). The patient was subsequently diagnosed with IOTB and started on standard four-drug anti-tubercular therapy (ATT). Four weeks after initiation of ATT and oral corticosteroids, there was a decrease in the vitritis and an improvement in BCVA to 20/60. The clarity of the media improved, and choroidal lesions appeared inactive on clinical examination and imaging. Ten weeks after initiation of ATT, the BCVA was 20/40 and the chorioretinal lesions stabilized without any further progression (Fig. 5). At her last follow-up visit (5 months after initiation of ATT), the lesions were healing well without any progression.

### Discussion

The index case presented as a diagnostic challenge since granulomatous panuveitis in a monocular patient with a history of trauma in the fellow eye was highly suggestive of sympathetic ophthamia. Our patient demonstrated interval improvement in vitritis and optic disc edema, and the choroidal lesions seemed to heal during the initial follow-up after initiation of intravenous methylprednisolone, oral corticosteroids, and azathioprine (Fig. 2a). However, as the patient was on continued immunosupression, her choroidal lesions increased in number and size, and the disease continued to progress. Thus, at this stage, an alternate diagnosis was considered. Multimodal imaging analyses were performed which revealed presence of distinct hypo-autofluorescent lesions with hyper-autofluorescent borders in the retinal periphery (Fig. 2e, f). The appearance of these lesions was not a characteristic of sympathetic ophthalmia [7]. Moreover, careful analysis of EDI-OCT scans passing through these peripheral choroidal lesions revealed presence of multiple, large choroidal granulomas which were different from those observed in sympathetic ophthalmia (Fig. 3) [8]. Since the fellow eye was non-seeing, the patient was given an option of enucleation to confirm an alternate diagnosis and conclusively rule out sympathetic ophthalmia.

An important learning point in our case was the detection of choroidal granulomas on EDI-OCT and identification of FAF lesions (Figs. 2 and 3). We hypothesize that immunosuppression in our patient led to development of a IOTB with multifocal disease in the form of multiple choroidal granulomas. Such a form of IOTB is rarely reported in literature. Due to the paucibacillary nature of the disease in a majority of the patients, nucleic acid amplication techniques are more commonly applied to confirm the diagnosis of IOTB. Only a handful of cases with histopathological detection of acid-fast bacilli from choroidal biopsies have been reported in the literature [9–11]. However, these were patients with large choroidal masses, subretinal abscesses, or those undergoing an unrelated retinal surgery. Thus, previous reports have highlighted the paucity of mycobacteria on histology in eyes with ocular TB [11]. The index case is unique because in our patient, the diagnosis of IOTB was possible from the histopathological analysis of the fellow non-seeing eye, and comprehensive multimodal imaging analysis of the affected eye. In addition, PCR analysis of the aspirate from the enucleated globe agreed with the histopathology, imaging, and laboratory evaluation (Fig. 4).

Thus, the diagnosis of IOTB is often challenging due to its protean clinical manifestations. However, once the diagnosis is confirmed, treatment with ATT and corticosteroids may result in resolution of the choroiditis lesions with progressive hypo-autofluorescence on FAF imaging. The limitations of our case were the non-availability of indocyanine green angiography to detect the choroidal granulomas. However, choroidal granulomas can be well demonstrated on EDI-OCT. [12, 13] Our patient demonstrated adequate response to ATT with gradual healing response of the choroiditis lesions, vitritis, and optic disc edema (Fig. 5).

### Conclusions

The index case report demonstrates challenges encountered in diagnosing and managing IOTB. Our patient presented with granulomatous panuveitis that was initially misdiagnosed as sympathetic ophthalmia. Techniques such as histopathology, multimodal imaging including FAF and EDI-OCT, as well as PCR analysis greatly aid in arriving at the accurate diagnosis in such challenging cases.
